# The Selection of the Optimal Impregnation Conditions of Vegetable Matrices with Iodine

**DOI:** 10.3390/molecules27103351

**Published:** 2022-05-23

**Authors:** Agata Zaremba, Katarzyna Waszkowiak, Dominik Kmiecik, Anna Jędrusek-Golińska, Maciej Jarzębski, Krystyna Szymandera-Buszka

**Affiliations:** 1 Department of Gastronomy Science and Functional Foods, Faculty of Food Science and Nutrition, Poznań University of Life Sciences, Wojska Polskiego 31, 61-624 Poznań, Poland; agata.zaremba@up.poznan.pl (A.Z.); katarzyna.waszkowiak@up.poznan.pl (K.W.); anna.jedrusek-golinska@up.poznan.pl (A.J.-G.); 2 Department of Food Technology of Plant Origin, Faculty of Food Science and Nutrition, Poznań University of Life Sciences, Wojska Polskiego 31, 61-624 Poznań, Poland; dominik.kmiecik@up.poznan.pl; 3 Department of Physics and Biophysics, Faculty of Food Science and Nutrition, Poznań University of Life Sciences, Wojska Polskiego 38/42, 60-637 Poznań, Poland; maciej.jarzebski@up.poznan.pl

**Keywords:** iodine, iodine carriers, fortification, minerals, vegetables

## Abstract

This study aimed to determine the use of selected vegetables (pumpkin, cauliflower, broccoli, carrot) as carriers of potassium iodide (KI) and potassium iodate (KIO_3_) by determining changes in iodine content under various conditions of impregnation as the degree of hydration, impregnated sample temperature, and impregnation time. The influence of these conditions on iodine contents in vegetables after their fortification and storage (21 °C/230 days) was analyzed. The results showed that all selected vegetables could be efficient iodine carriers. However, the conditions of the impregnation process are crucial for fortification efficiency, particularly the degree of hydration and the temperature of the impregnated samples before drying. The results showed that the lowest iodine content was in samples fortified at 4 °C and 1:4 hydration. On the other hand, the highest reproducibility of iodine was for the following fortification conditions: temperature of −76 °C and hydration of 1:1. The studies confirmed the higher stability of iodine in KIO_3_ form compared to KI. To increase recovery of the introduced iodine in the product after drying, using the conditioning step at 4 °C is not recommended. We recommend freezing vegetables immediately after the impregnation process

## 1. Introduction

Nutrient deficiencies are common in developing countries, but they are also found in developed ones [1,2]. Iodine deficiency is one of the most common nutrient deficiencies [3,4]. The crucial cause of iodine deficiency is low levels of iodine in foods [5]. The daily requirement for iodine is 150 mcg/day. Food enrichment offers significant benefits, from reducing the prevalence of nutritional deficiencies to providing benefits for societies and economies [6,7,8,9,10,11].

The possible forms of food enrichment include agricultural strategies of biofortification of plants with nutrients, such as iron, iodine, zinc, or folic acid [12]. These methods aim to enrich consumers’ diets with nutrients, including iodine, by increasing the concentration of a particular element in the edible part of the plant before harvesting [12,13,14]. Pilot studies confirm that it is possible to use iodine for the biofortification of vegetables [4,15]. The undoubted advantage of agronomic biofortification is the possibility of increasing the content of this element in plants during their natural growth [7,16]. However, biofortification with iodine can also affect the content of other minerals. These changes can be negative, as they can reduce the plant’s nutrition with other important micro- and macro-nutrients, thereby reducing the nutrient content of plant food products. Biofortification of plants with iodine is a relatively time-consuming method, requiring yield quality control at many stages of plant growth, thus generating additional costs [17].

Another method of enrichment is to introduce nutrients during the production process after harvesting the plant. This method is considered to be the most effective and cost-efficient way to prevent mineral and vitamin deficiencies, even safer than supplementation [4,18,19].

Enriching foods with iodine may be another way to eliminate iodine deficiency, usually by table salt iodization [20,21,22,23,24]. Potassium iodide and potassium iodate are sources of iodine in table salt. Table salt iodization is used as an intervention fortification in many countries, including Poland [3,23]. However, in 2006 the World Health Organisation introduced a recommendation to limit salt intake to 5 g/d as it is a risk factor for atherosclerosis and hypertension. It is, therefore, necessary to find new carriers for iodine salts [25,26,27].

Previous studies have confirmed the feasibility of applying iodine salts (KI and KIO_3_) to protein preparations such as collagen, soy preparations, and fiber preparations. Methods for the enrichment of protein preparations have already been developed [28,29,30,31]. Nevertheless, high protein intake is not advisable in some diets [32]. Consumers may also show little confidence in high-protein preparations, associating them with the diets of physically active people rather than as a standard part of everyday diet [33,34,35]. The application of protein preparations, especially soy, may be limited for allergy sufferers [36].

The fortification of vegetables may constitute an attractive alternative source of iodine for all groups of consumers, especially vegetarians and vegans [37,38,39,40]. The selection of plant-based products also allows for an increase in the dietary intake of fiber, for which dieticians recommend increasing daily amounts in an adult’s diet to 25–35 g [41,42].

Food fortification is a relatively simple and highly effective method of preventing and treating the most common nutrient deficiencies in the population, including iodine deficiency [6,18,43]. Economically and politically, it is quite profitable and is easily accepted by society [40,44]. It can be nationally standardized and controlled. However, it should be noted that the effectiveness of a food fortification program is measured based on the improvement of the nutritional and health status of the target population [6,45]. This result can be achieved, among other things, by identifying the critical points of the adopted fortification stages [46,47]. Therefore, when designing a food fortification program, it is necessary to identify the optimum impregnation conditions related to the choice of the nutrient form, matrix, variable conditions of fortification, water removal as well as storage to maximize the content of the nutrient in the final product [48,49]. This will make it possible to identify the maximum shelf life for the product under specified conditions and to indicate it correctly on the label.

The stability of nutrients is related to conditions of application of the nutrient to the matrix [20,30,50,51,52]. Iodate is a strong oxidizing agent, and iodide is a reducing agent, which can lead to the initiation of redox reactions in foods. These reactions may be, in turn, associated with changes in the stability and shelf life of food ingredients [30,53,54]. Iodine is also sensitive to light and temperature [55]. During the fortification process, it is crucial to determine the optimum time and hydration [56] of the matrix to achieve even distribution and, at the same time, stability of the introduced compound during processes related to the storage or production of new food products [30,48,55]. Therefore, this study aimed to investigate the use of selected vegetables (pumpkin, cauliflower, broccoli, carrot) as carriers of potassium iodide (KI) and potassium iodate (KIO_3_) by determining changes in the iodine content under various conditions of impregnation with iodine compounds. It was also hypothesized that the fortification parameters affect the continued stability of the applied iodine during the storage of the enriched dried product. Our study assessed the impact of the treatment after the impregnation process. It was investigated whether a conditioning step of the fortified plant material at 4 °C was required before freeze-drying or if it was better to freeze it immediately for the process. The influence of the freezing temperature on iodine retention (−21 °C and −76 °C) was also investigated.

## 2. Results and Discussion

### 2.1. L*a*b* Color Properties

The tables containing all the color parameters data are included in Supplementary Materials Tables S1–S8. It was found that the application of iodine to the analyzed vegetables did not affect their color parameters (L*a*b*). Additionally, the application of variable impregnation conditions did not alter these parameters (Tables S1–S8). It was confirmed that regardless of the variant of the fortification process used (hydration, temperature, and time) within the same vegetables, the differences were statistically insignificant.

### 2.2. Iodine Content after Fortification

**Figure 1:**
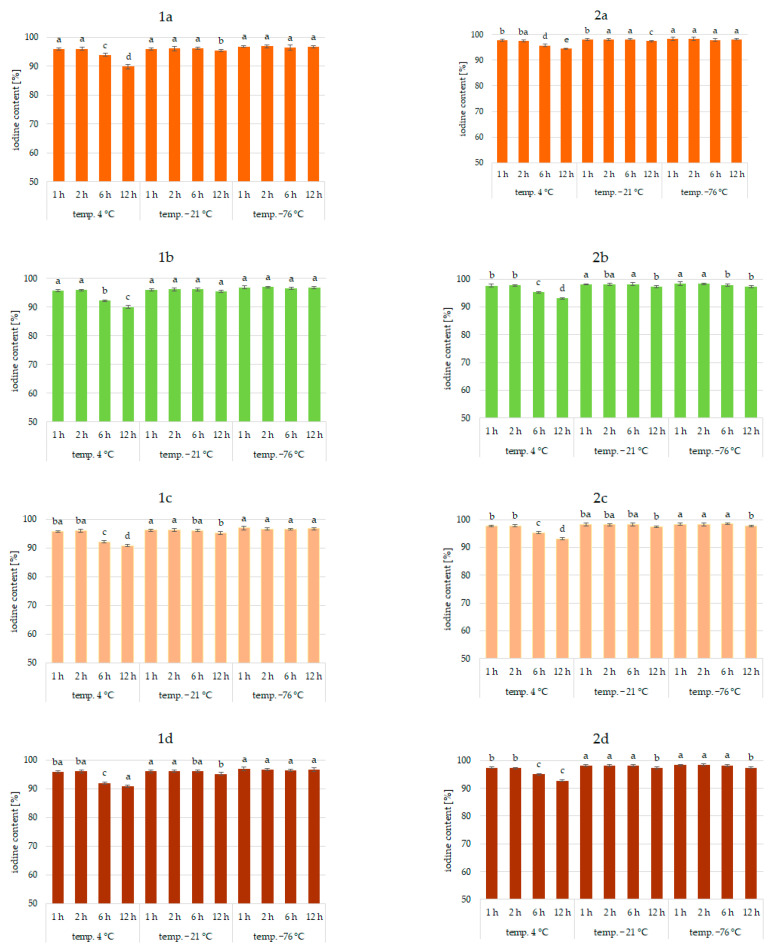
Iodine content (%) in pumpkin (**1a**,**2a**), broccoli (**1b**,**2b**), cauliflower (**1c**,**2c**), and carrot (**1d**,**2d**) fortified with KI and KIO_3_, respectively, at hydration ratio 1:1 and different temperatures (4 °C, −21 °C, −76 °C) and times; different letters (a–e) denote a significant difference at *p* < 0.05 (one-way ANOVA, and post hoc Tukey test). Mean values (*n* = 4). Error bars are confidence intervals with a confi-dence coefficient of 0.95.

**Figure 2:**
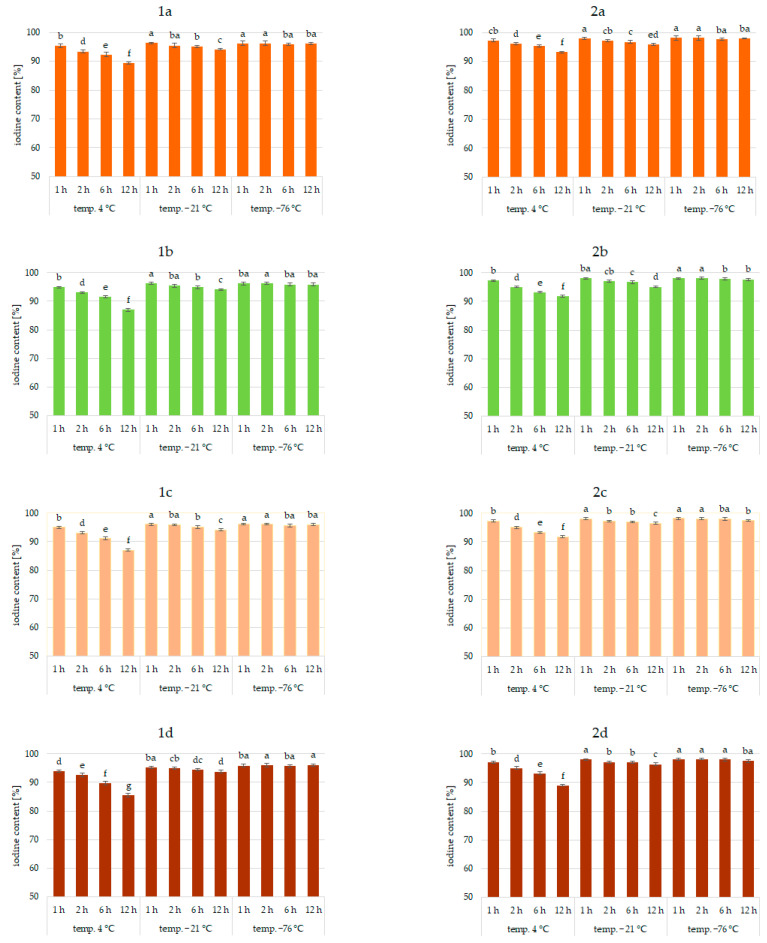
Iodine content (%) in pumpkin (**1a**,**2a**), broccoli (**1b**,**2b**), cauliflower (**1c**,**2c**), and carrot (**1d**,**2d**) fortified with KI and KIO_3_, respectively, at hydration ratio 1:2 and different temperatures (4 °C, −21 °C, −76 °C) and times; different letters (a–g) denote a significant difference at *p* < 0.05 (one-way ANOVA, and post hoc Tukey test). Mean values (*n* = 4). Error bars are confidence intervals with a confidence coefficient of 0.95.

**Figure 3:**
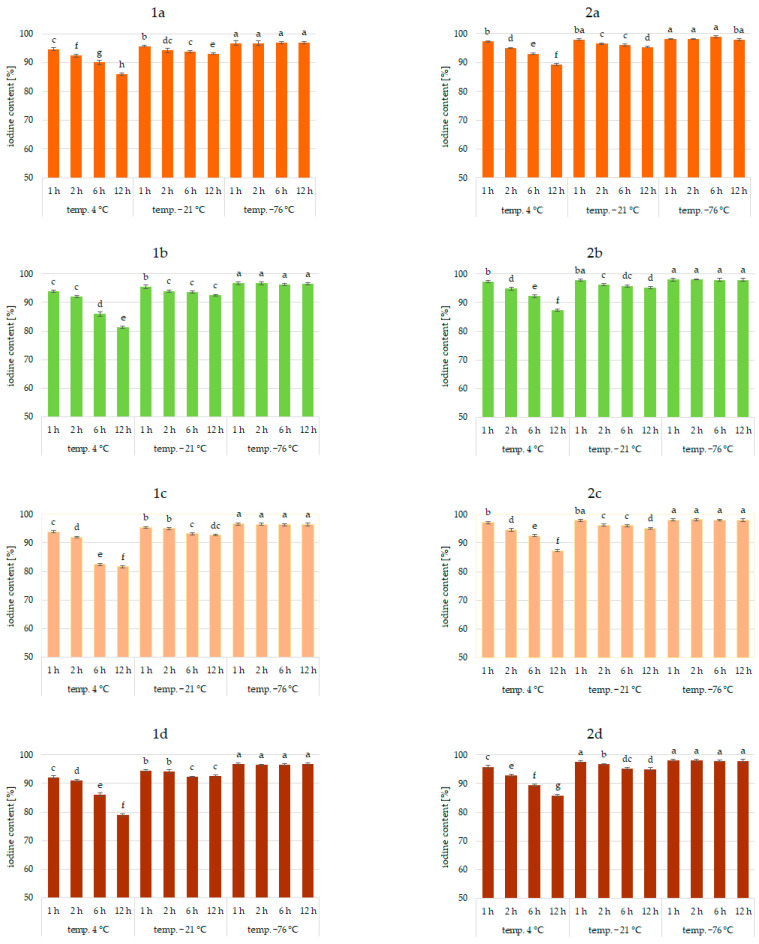
Iodine content (%) in pumpkin (**1a**,**2a**), broccoli (**1b**,**2b**), cauliflower (**1c**,**2c**), and carrot (**1d**,**2d**) fortified with KI and KIO_3_, respectively, at hydration ratio 1:4 and different temperatures (4 °C, −21 °C, −76 °C) and times; different letters (a–h) denote a significant difference at *p* < 0.05 (one-way ANOVA, and post hoc Tukey test). Mean values (*n* = 4). Error bars are confidence intervals with a confidence coefficient of 0.95.

Results of the study confirmed the effectiveness of the application of vegetables as a matrix for iodine application. This was true for all analyzed vegetables and the forms of iodine (KI, KIO_3_). Figure 1, Figure 2 and Figure 3 show the iodine content (%) of enriched vegetables (pumpkin, broccoli, cauliflower, and carrot) after the drying process of samples fortified with iodine KI and KIO_3_ using variable parameters of fortification, i.e., temperature (4, −21, −76 °C) and time (1, 2, 6, 12 h) of conditioning, at three hydration conditions (1:1, 1:2, and 1:4, respectively). 

An analysis of iodine content (Figure 1, Figure 2 and Figure 3) showed a recovery of the introduced iodine in the product after drying to 98%. This level can be considered very high [30,57,58]. Previous data on the fortification of protein preparations confirm the maximum reproducibility of iodine in fortified matrices at a similar level. However, there was considerable variability in the results related to the variable parameters of iodine fortification.

The differences in iodine content in the range of 78–97% were confirmed, depending on the selected fortification method. The lowest iodine content (78%) was found in KI-enriched carrot samples fortified after 1:4 hydration at 4 °C for 6 h. The highest reproducibility of iodine was found when the pumpkin matrix was fortified using KIO_3_ at the temperature of −76 °C and 1:1 hydration conditions.

The analysis of covariance showed (Table 1) a statistically significant effect (*p* < 0.05) of the type of iodine compound used for fortification (KI, KIO_3_). However, taking all predictive factors into account (Table 1), the impregnation temperature, followed by the degree of hydration associated with iodine application, was confirmed to have a stronger effect on the final iodine content than the form of iodine.

It was found that the total iodine content after drying the samples was most strongly correlated with the degree of hydration (r = 0.903) and the temperature of the impregnated samples (r = 0.99). This is also confirmed by previous studies [30,56,59,60]. The lowest stability of iodine was shown for samples impregnated at 4 °C. This phenomenon can be explained by the fact that the higher the temperature, the higher the rate of conversion of iodine compounds, especially potassium iodide. A higher rate of conversion of iodine compounds to free iodine is related to the lower activation energy required for this process [61]. All unfrozen water is maintained under these conditions, resulting in increased activity of the iodine compounds dissolved in a solution. In addition, in a non-frozen structure, the passage of iodine to the atmosphere through diffusion is much more efficient [62]. The lower the temperature of the samples, the lower the activity of this process [54,59,63].

The lowest iodine content (79%) was in samples impregnated with 1:4 hydration. Regardless of the impregnation temperature, the iodine content of the sample was lower by 11% compared to samples impregnated at 1:1 hydration. This fact could be attributed to the accelerating chemical reactions of KI and KIO_3_ with increasing water content in the system and decreasing activation energy for the conversion of iodine compounds and evaporation [59,62,64]. The highest differences (11%) were observed for samples impregnated at an impregnation temperature of 4 °C. Using an impregnation temperature of −76 °C, iodine losses at 1:2 hydration were only 4% higher on average, and at 1:1 were statistically insignificant. This fact is confirmed by the increase in activity at 4 °C.

Impregnation time was also a factor affecting iodine content (r = 0.765). However, the significance of this factor depended on the degree of hydration and even more on the impregnation temperature [30,55,60,62,65]. The use of 1:4 hydration and impregnation temperature of 4 °C yielded total iodine ranging from 78 to 97%. The lowest iodine content (78%) was in samples fortified at 4 °C for 12 h.

For an incubation temperature of −21 °C and hydration of 1:4, the content of total iodine in samples fortified for 12 h was higher by 6–7% compared to 4 °C, and for the hydration of samples of 1:2, by 6–9%. The effect of incubation time for the impregnation temperature of −76 °C was statistically insignificant. These trends concerning the effect of impregnation time are confirmed by previous studies indicating that prolonged exposure of components to adverse factors increases iodine loss [59,61,64]. Increased time at conditions of the highest potassium iodide and potassium iodate activity promotes the transition to free iodine forms and facilitates volatilization [20,57,59].

When analyzing the form of iodine, KI showed higher sensitivities to impregnation conditions, which was especially true for 1:4 hydration and a fortification temperature of 4 °C. Differences varied up to 12%. Previous studies also confirmed the higher instability of iodine in the form of KI [30,53,54]. Iodine in this form is easily sublimed and then quickly lost to the atmosphere by diffusion. The lower activation energy of this process compared to iodate transformations can explain this. Potassium iodate can be reduced by agents present in the carrier, e.g., iron ions or environmental reaction, but the process is longer. An increase in temperature and water content increased the rate of both reactions [61,64].

Significant differences in the iodine content of the samples depending on the type of vegetable were not found. The only exception was impregnation conditions of 1:4 hydration, fortification temperature of 4 °C, and an impregnation time of more than 6 h, where a significant effect of the type of enriched vegetable was found (r > 0.985). The highest losses were confirmed for samples of carrots and the lowest for pumpkin. The highest stability of iodine applied to pumpkins may be related to the higher protein content and lower pH of the product compared to carrots [66,67]. This is confirmed by previous data on the impregnation of vegetables with thiamine [42].

#### Principal Component Analysis (PCA)

**Figure 4:**
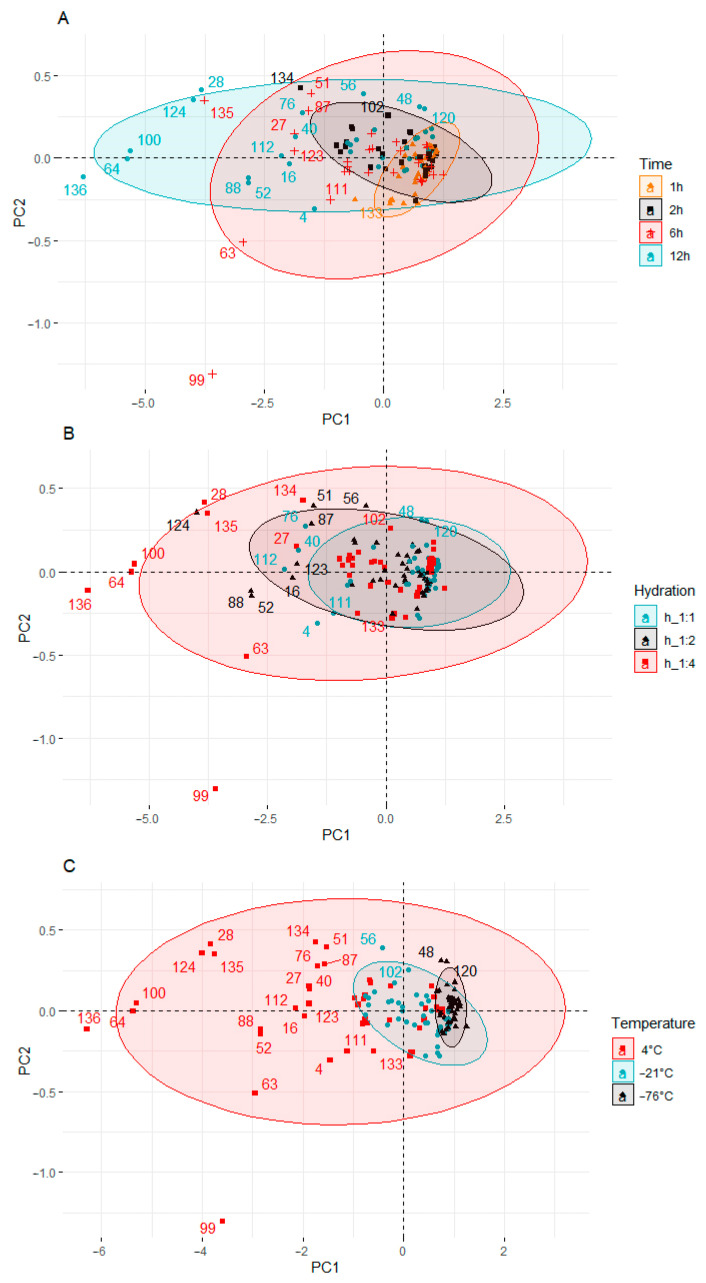
Principal component analysis (PCA) of the score plot of data from KIO_3_ and KI content in pumpkin, broccoli, cauliflower, and carrot fortified at different times (**A**), hydration ratios (**B**), and temperatures (**C**).

Principal component analysis (PCA) was applied to observe possible clusters in fortified pumpkin, broccoli, cauliflower, and carrot prepared at different times, hydration degrees, and temperatures. The result of the distribution of the samples depending on the differentiating factor (time, hydration degree, and temperature) is shown in Figure 4.

Four clusters were found (Figure 4A) when time effect was analyzed. Samples fortified 1 and 2 h are located close to the plot center, not far from each other. The exception to this is a cauliflower sample fortified for 2 h at 4 °C and 1:4 hydration (134). This sample had the lowest content of KIO_3_ and KI among the samples fortified for 2 h.

In the two remaining groups, a much greater dispersion of the samples was observed. When the fortification time was 6 h, most of the samples were on the left side of the y-axis. Two outliers were also observed in this group: broccoli (63) and cauliflower (99) samples, which were fortified at 4 °C and 1:4 hydrations. Both samples were below the x-axis. The samples fortified for 12 h showed the highest dispersion. All samples in this group were distributed along the y-axis.

When analyzing hydration degree, a much larger dispersion of the samples was observed (Figure 4B) compared to the plot when the differentiating factor was time. In the first two groups (hydration 1:1 and 1:2), the dispersion scale of the samples was similar. When the hydration was 1:4, many outliers outside the group of samples located near the center of the plot could be observed. The outliers, apart from the highest hydration, were also characterized by the longest fortification time (12 h) carried out at the highest temperature (4 °C).

When the process was carried out at −76 °C, the content of KIO_3_ and KI was the highest, and the samples were the least diverse. This group of samples is located to the right of the y axis and at a small distance from each other. The KIO_3_ and KI content ranged from 2.4847 to 2.5246 (mg DM I kg^−1^) and from 2.4449 to 2.4786 (mg DM I kg^−1^), respectively. When the temperature was higher (−21 °C), the samples were still well grouped, but their dispersion was bigger. The samples were shifted to the left towards the center of the graph. In samples prepared at −21 °C, the content of KIO_3_ and KI was lower and ranged from 2.4280 to 2.5093 (mg DM I kg^−1^) and from 2.3585 to 2.4600 (mg DM I kg^−1^), respectively. The highest dispersion of the results was characteristic of the process carried out at the temperature of 4 °C. In this cluster, there were samples with both the highest and the lowest content of KIO_3_ and KI.

The factor that most influenced the diversity of the groups was the temperature of the fortification process (Figure 4C). There are three clusters in the score plot.

The PCA results showed differences between the individual vegetable samples fortified under different conditions of time, temperature, and hydration. The samples prepared under the conditions of 1 h, 1:1 hydration ratio, and temperature of −76 °C were characterized by the lowest variability and the highest KIO_3_ and KI content. The extension of the processing time, the increase in the degree of hydration, and the temperature were related to the decrease in the content of iodine compounds in the analyzed samples. The differentiation of the samples resulted to a greater extent from the process conditions than from the type of vegetable used.

### 2.3. Storage of Iodine Sources

This study showed the significant effect of the impregnation conditions on the stability of iodine during storage. The tables containing all the iodine concentration data are included in the Supplementary Materials Tables S9–S16. The experiment assumed storage at 21 °C. Analysis of the dynamics of changes in iodine content (half-life T_(1/2)_) based on the adopted model (Table 2) [30] showed that all variable impregnation conditions, i.e., temperature, degree of hydration, and time, significantly affected the stability of iodine in vegetable matrices. Iodine content (% relative to the amount after drying) of fortified vegetables after 230 days of storage differed, ranging from 83 to 67% (Tables S9–S16). The stability of iodine in the vegetable carriers during storage was at least as high as in protein and fiber carriers and higher than in table salt shown in previous studies [30,54,65,68].

It was confirmed that the most unfavorable iodine impregnation conditions decreased iodine stability the most during sample storage (Table 2).

This was especially true for KI, for which the rate of iodine loss was faster, up to 11%, compared to KIO_3_. This observation is explained by the lower stability of KI and a higher rate of iodine transformation transition to free iodine during impregnation [56].

It was found that the total iodine content after the storage (230 days) of dried fortified samples was correlated most strongly with the degree of hydration (r = −0.801), especially for impregnation at 4 °C (r = −0.972). Iodine losses were the lowest for samples impregnated at −76 °C (22–24%) and highest for those impregnated at 4 °C (27–32%). The half-life values (Table 2) suggest that the lower the impregnation temperature and the degree of hydration of the vegetables, the lower the dynamics of iodine loss during dried storage. For samples fortified at −21 °C, the iodine losses were higher compared to −76 °C (by an average of 10%) and lower than at 4 °C (by about 7%).

The rate of iodine transformation during storage was the highest for samples impregnated at 1:4 hydration and 4 °C, irrespective of the form of iodine applied or matrix. For samples impregnated at the hydration degree of 1:4 and the temperature of 4 °C, the incubation time was also a relevant factor affecting losses during sample storage. Increasing the incubation time to 12 h resulted in a reduction in T_(1/2)_ of iodine up to 20% (1:4) and 9% (1:2) during storage. At lower temperatures (−76 °C), the impregnation time did not affect its stability.

The least significant factor affecting the stability of applied iodine during storage was the type of vegetable. The vegetable matrix only influenced iodine stability in stored vegetable samples pre-impregnated at 1:4 hydration and 4 °C. The half-lives of iodine T_(1/2)_ for pumpkin impregnated in these parameters were longer, up to 20%, compared to the samples of carrot. The ranking of vegetables according to increasing sensitivity to changing conditions of pre-impregnation during storage was as follows: pumpkin > cauliflower = broccoli > carrot.

Further research on the correlation between the type of vegetable carriers (especially pumpkin and carrot) and the stability of iodine is necessary to clarify this point. The results may be interesting for nutritionists, as well as for food producers who offer food for consumers at risk of iodine deficiency, for example, vegans and vegetarians.

## 3. Materials and Methods

### 3.1. Material

Vegetables, i.e., pumpkin (*Cucurbita pepo* L.), cauliflower (*Brassica oleracea* var. *botrytis* L.), broccoli (*Brassica oleracea* L.), and carrot (*Daucus carota* L.), were used as a matrix for the iodine. The products in a ripe state were purchased in the retail trade in the months of September and October. The KI and KIO_3_ constituted the sources of iodine (Merck, Darmstadt, Germany).

#### 3.1.1. Conditions of Impregnation

**Figure 5:**
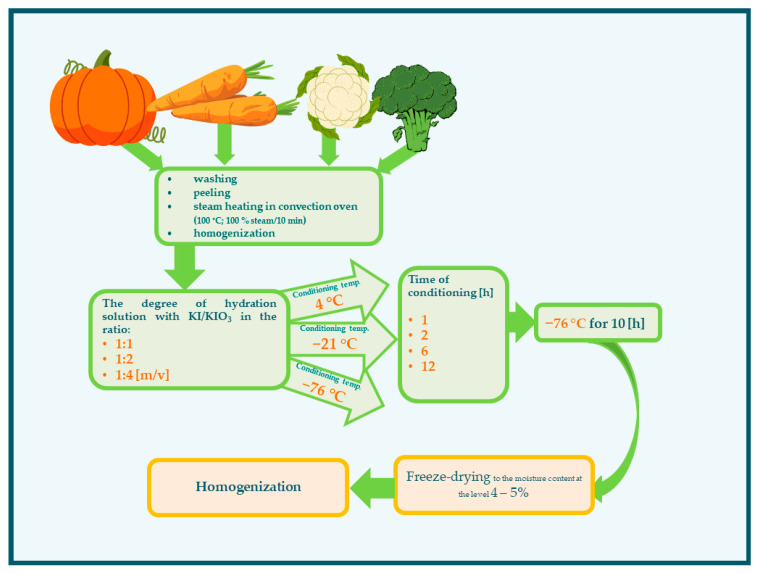
Scheme of the impregnation conditions of vegetables with iodine.

The experimental scheme is presented in Figure 5. The vegetables were washed under running tap water. The pumpkin and carrot were peeled with knives, and the seeds of the pumpkin were removed. The vegetables were cut into small pieces: pumpkins into cubes in size about 4 × 4 × 4 cm, carrots in slices 4 cm thick, and cauliflower and broccoli in florets. Next, the vegetables were steamed (100 °C; 100% steam/10 min) in a convection oven (Rational, Landsberg am Lech, Germany). The vegetables were subsequently drained and subjected to homogenization (homogenizer—Foss, Hilleroed, Denmark) to obtain a particle size of 250 µm. The next stage of impregnation was the conditioning of the vegetables in an aqueous solution of KI/KIO_3_ (Table 3). For the next impregnation steps, the variability of conditions was assumed:

- Degree of hydration—in the ratio 1:1, 1:2, 1:4 (*m*/*v*) at temperature 21 °C;
- Temperature of conditioning (4, −21, −76 °C);
- Time of conditioning (1, 2, 6, 12 h).

All the samples (including those impregnated at −76 °C) were stored at −76 °C for 10 h before drying. Then, the impregnated preparations were freeze-dried (Alpha 1–4 443 LSC Freeze dryer; Christ, Hagen, Germany; at the temperature of the shelf 14 °C and condenser −54 °C, and vacuum 0.520 mbar) to the moisture content at the level of 4–5%. Freeze-drying times for samples hydrated in the ratio 1:1 were 28–30 h, and 1:3 and 1:4 were 49–53 h and 69–74 h, respectively. The dried vegetables were subjected to homogenization (homogenizer—Foss, Hilleroed, Denmark) to obtain a powder particle size of approximately 250 µm.

#### 3.1.2. Storage Conditions of Iodine Sources

The impregnated and freeze-dried vegetables under investigation were stored in jars (black glass, closed with screw top, d = 7 cm, h = 10 cm). The influence of storage conditions on the stability of KI and KIO_3_ was tested during storage of 21 ± 1 °C.

The iodine contents in the investigated carriers were monitored on the selected storage days: 1, 30, 60, 90, 120, 150, 180, and 230.

### 3.2. Methods

#### 3.2.1. Stability of Iodine

In order to determine the effectiveness of the iodine impregnation conditions, the iodine content of the vegetables was determined after the application of iodine and storage.

Directly after drying of fortified samples, quantitative changes in the total and inorganic iodine were determined with a macro chemical method with potassium thiocyanate described by Kuhne, Wirth, and Wagner [69] and Moxon and Dixon [70]. The details of the method were described previously by Waszkowiak and Szymandera-Buszka [71].

#### 3.2.2. Dry Mass

Iodine content was converted to dry weight. For this purpose, the dry mass (DM) of iodine carriers was estimated by drying at 105 °C to constant weight [72].

#### 3.2.3. L*a*b* Color Properties

For L*, a*,b* color evaluation of powdered vegetables, an NH310 portable colorimeter (Shenzhen Three NH Technology Co., Ltd., Shiyan, China) equipped with Light Source is LED blue light excitation with illuminating/viewing geometry 8/d, and internal software was applied. Before the measurements, the colorimeter was calibrated with the use of a white plate (provided by the manufacturer), and the black color calibration was manually performed (calibration on air). During measurements, the powder of the vegetables was inserted into the dedicated powder dark chamber.

The color tests were repeated 10 times, and average values with SD were recorded [73].

### 3.3. Statistical Analysis

STATISTICA PL 13.3 (Tibco Software Inc., Palo Alto, CA, USA) and R software (version 4.1 with packages FactoMineR v.2.4 and factoextra v.1.0.7) were the software used for principal components analysis (PCA) and calculating significant differences between means (*p* < 0.05, analysis of variance ANOVA), Tukey’s multiple range test.

The iodine content of the tested samples was analyzed in 6 samples (2 independent samples and 3 measurements for each sample). Hypotheses were tested at α = 0.01. To predict the dynamics of changes in iodine content in carriers during storage, the half-life value (T_1/2_) was used. This is a term that describes the time within which the initial iodine content decreases by half. The half-life was calculated from an exponential decay mode [30]. The accuracy of the models was estimated using the coefficient of determination (R^2^) and root mean square error (RMSE). The significance level for all analyses was set at 5%.

## 4. Conclusions

The research presented in this paper shows that all selected vegetables (pumpkin, broccoli, cauliflower, and carrots) can be used as iodine carriers and can therefore be an alternative to table salt and even protein preparations. The presence of introduced iodine did not affect the color parameters of the dry vegetables fortified with iodine.

The results confirmed the hypothesis that the fortification parameters affect the stability of the iodine during fortification and continued storage.

After drying the samples, the total iodine content was most strongly correlated with the degree of hydration and the temperature of the impregnation process. The lowest iodine content was in samples fortified with 1:4 hydration at 4 °C for 6 h. The highest reproducibility of iodine was for the following fortification conditions: temperature of −76 °C and 1:1 hydration.

This study confirms that the most unfavorable iodine impregnation conditions also decreased iodine stability during storage of the fortified samples. The preferred form of iodine was KIO_3_ rather than KI.

In order to maximize recovery of the introduced iodine in the product after drying up, using the conditioning step at 4 °C is not recommended. On the other hand, freezing fortified vegetables immediately after the impregnation process is strongly recommended.

## Figures and Tables

**Table 1:** Statistical significance of predictors of covariance models for changes in iodine content in selected iodine carriers during impregnation.

Predictors	SS	df	SEM	F-Value	*p*-Value	Post hoc Power α = 0.05
Temperature	0.676	2	0.338	80.00	0.000	1.00
Hydration	0.125	2	0.063	10.20	0.000	0.98
Time	0.269	3	0.090	15.80	0.000	0.99
Form iodine	0.253	1	0.253	44.60	0.000	0.99
Vegetable	0.017	3	0.006	0.80	0.473	0.23

SS—Statistical Significance; df—degrees of freedom SEM—the Standard Error of the Mean.

**Table 2:** Dynamic of changes in iodine content (mg kg^−1^) during 230 days of storage of the dried iodine fortified vegetables under various conditions (temperature, hydration, and time).

Fortification Parameters			Dynamic of Change in Iodine Content during 230 Days									
Hydratation	Temperature	Time [Hours]	T_1/2_ (Days)	*R* ^2^	*RMSE*	k	A_0_	T_1/2_ (Days)	*R* ^2^	*RMSE*	k *	A_0_ *
			KIO3					KI				
PUMPKIN												
1:1	4 °C	1	344	0.988	0.016	−0.00202	12.30	291	0.988	0.019	−0.00239	11.57
		2	341	0.983	0.019	−0.00203	12.21	292	0.990	0.017	−0.00238	11.54
		6	349	0.991	0.014	−0.00199	11.54	289	0.982	0.023	−0.00240	10.95
		12	349	0.988	0.016	−0.00199	11.09	301	0.987	0.019	−0.00230	9.75
	−21 °C	1	358	0.976	0.112	−0.00194	12.32	294	0.993	0.359	−0.00236	11.58
		2	360	0.977	0.021	−0.00193	12.37	292	0.989	0.018	−0.00237	11.64
		6	355	0.971	0.025	−0.00195	12.42	294	0.992	0.016	−0.00235	11.62
		12	372	0.982	0.030	−0.00187	12.13	297	0.992	0.304	−0.00234	11.44
	−76 °C	1	327	0.982	0.021	−0.00212	12.62	291	0.993	0.031	−0.00238	11.81
		2	328	0.982	0.021	−0.00211	12.60	297	0.992	0.015	−0.00233	11.79
		6	328	0.985	0.021	−0.00211	12.49	293	0.992	0.016	−0.00237	11.75
		12	325	0.983	0.021	−0.00213	12.56	296	0.994	0.016	−0.00234	11.73
1:4	4 °C	1	275	0.977	0.028	−0.00252	12.33	275	0.991	0.010	−0.00273	11.18
		2	271	0.966	0.035	−0.00256	11.72	251	0.984	0.010	−0.00276	10.63
		6	288	0.979	0.026	−0.00241	10.80	267	0.981	0.015	−0.00260	9.72
		12	278	0.969	0.032	−0.00249	9.81	261	0.985	0.017	−0.00265	8.79
	−21 °C	1	281	0.964	0.376	−0.00247	12.76	262	0.984	0.254	−0.00264	11.65
		2	294	0.966	0.032	−0.00236	12.17	276	0.975	0.005	−0.00251	11.13
		6	302	0.964	0.032	−0.00230	11.75	278	0.969	0.011	−0.00250	10.75
		12	306	0.975	0.026	−0.00227	11.41	279	0.974	0.013	−0.00248	10.45
	−76 °C	1	328	0.983	0.115	−0.00211	12.55	293	0.994	0.303	−0.00236	11.73
		2	325	0.980	0.020	−0.00214	12.55	294	0.993	0.002	−0.00235	11.72
		6	330	0.983	0.021	−0.00210	12.78	290	0.996	0.002	−0.00239	11.79
		12	326	0.984	0.022	−0.00212	12.47	290	0.993	0.001	−0.00239	11.84
BROCCOLI												
1:1	4 °C	1	324	0.977	0.024	−0.00214	12.27	290	0.976	0.027	−0.00239	11.55
		2	323	0.965	0.030	−0.00215	12.39	284	0.982	0.024	−0.00244	11.63
		6	333	0.983	0.020	−0.00208	11.36	298	0.983	0.022	−0.00233	10.38
		12	331	0.984	0.019	−0.00210	10.70	300	0.984	0.021	−0.00231	9.74
	−21 °C	1	332	0.967	0.028	−0.00209	12.38	290	0.981	0.365	−0.00239	11.51
		2	322	0.952	0.035	−0.00215	12.40	296	0.986	0.020	−0.00234	11.48
		6	332	0.964	0.029	−0.00209	12.41	297	0.989	0.018	−0.00233	11.50
		12	331	0.967	0.028	−0.00209	12.08	302	0.980	0.299	−0.00229	11.19
	−76 °C	1	335	0.963	0.035	−0.00207	12.30	292	0.986	0.041	−0.00237	11.66
		2	334	0.970	0.030	−0.00208	12.36	299	0.980	0.021	−0.00232	11.62
		6	326	0.966	0.030	−0.00213	12.35	298	0.983	0.021	−0.00232	11.53
		12	331	0.969	0.026	−0.00209	12.10	293	0.984	0.024	−0.00236	11.63
1:4	4 °C	1	261	0.965	0.037	−0.00266	12.58	304	0.990	0.017	−0.00228	10.84
		2	263	0.975	0.031	−0.00264	11.68	313	0.990	0.016	−0.00221	10.34
		6	275	0.982	0.025	−0.00252	10.64	286	0.989	0.036	−0.00242	8.79
		12	271	0.982	0.025	−0.00256	9.33	223	0.985	0.029	−0.00310	7.72
	−21 °C	1	270	0.973	0.394	−0.00257	12.75	398	0.991	0.344	−0.00174	11.34
		2	284	0.982	0.024	−0.00244	12.07	397	0.991	0.253	−0.00175	10.92
		6	297	0.978	0.025	−0.00233	11.70	290	0.989	0.018	−0.00239	10.71
		12	266	0.968	0.035	−0.00261	11.61	265	0.989	0.020	−0.00261	10.41
	−76 °C	1	317	0.978	0.127	−0.00218	12.49	332	0.991	0.175	−0.00209	11.65
		2	312	0.979	0.024	−0.00222	12.51	329	0.990	0.015	−0.00210	11.65
		6	311	0.977	0.024	−0.00223	12.48	337	0.991	0.015	−0.00206	11.56
		12	308	0.980	0.024	−0.00225	12.49	339	0.991	0.015	−0.00205	11.63
CAULIFLOWER												
1:1	4 °C	1	335	0.988	0.017	−0.00207	12.23	289	0.976	0.027	−0.00240	11.54
		2	335	0.984	0.019	−0.00207	12.32	289	0.982	0.023	−0.00239	11.57
		6	339	0.992	0.013	−0.00205	11.45	298	0.974	0.028	−0.00232	10.37
		12	340	0.992	0.013	−0.00204	10.72	297	0.986	0.020	−0.00234	9.92
	−21 °C	1	341	0.979	0.021	−0.00203	12.37	292	0.982	0.363	−0.00238	11.47
		2	339	0.977	0.023	−0.00204	12.34	294	0.979	0.025	−0.00236	11.46
		6	341	0.986	0.018	−0.00204	12.30	289	0.983	0.023	−0.00240	11.47
		12	342	0.987	0.017	−0.00202	12.05	293	0.981	0.308	−0.00237	11.18
	−76 °C	1	343	0.987	0.028	−0.00202	12.31	292	0.984	0.048	−0.00237	11.65
		2	335	0.979	0.017	−0.00207	12.45	297	0.979	0.022	−0.00233	11.50
		6	342	0.983	0.018	−0.00203	12.45	296	0.984	0.024	−0.00234	11.47
		12	340	0.981	0.022	−0.00204	12.22	289	0.984	0.025	−0.00240	11.58
1:4	4 °C	1	266	0.973	0.031	−0.00260	12.42	252	0.991	0.019	−0.00275	10.97
		2	263	0.975	0.031	−0.00263	11.62	246	0.989	0.022	−0.00281	10.55
		6	274	0.982	0.025	−0.00253	10.70	279	0.981	0.067	−0.00249	8.09
		12	271	0.982	0.025	−0.00255	9.29	261	0.974	0.033	−0.00266	7.91
	−21 °C	1	269	0.971	0.395	−0.00258	12.73	258	0.990	0.413	−0.00269	11.58
		2	275	0.976	0.029	−0.00252	12.11	256	0.981	0.263	−0.00271	11.43
		6	293	0.984	0.022	−0.00237	11.75	270	0.977	0.029	−0.00256	10.63
		12	284	0.972	0.030	−0.00244	11.55	268	0.980	0.027	−0.00259	10.43
	−76 °C	1	317	0.984	0.106	−0.00219	12.42	281	0.987	0.134	−0.00246	11.73
		2	315	0.986	0.020	−0.00220	12.44	287	0.993	0.021	−0.00241	11.68
		6	314	0.978	0.020	−0.00221	12.39	285	0.994	0.021	−0.00243	11.69
		12	317	0.986	0.019	−0.00219	12.30	278	0.992	0.014	−0.00249	11.73
CARROT												
1:1	4 °C	1	311	0.983	0.022	−0.00223	12.13	279	0.980	0.026	−0.00249	11.43
		2	309	0.982	0.022	−0.00224	12.07	279	0.985	0.023	−0.00248	11.40
		6	309	0.990	0.016	−0.00224	11.38	291	0.976	0.027	−0.00238	9.96
		12	312	0.989	0.017	−0.00222	10.65	291	0.985	0.022	−0.00238	9.43
	−21 °C	1	315	0.981	0.022	−0.00220	12.36	284	0.983	0.372	−0.00244	11.09
		2	312	0.974	0.026	−0.00222	12.32	285	0.979	0.026	−0.00243	11.05
		6	306	0.983	0.021	−0.00226	12.29	279	0.983	0.024	−0.00248	11.28
		12	306	0.986	0.020	−0.00227	12.05	283	0.981	0.319	−0.00245	11.02
	−76 °C	1	293	0.991	0.028	−0.00237	12.40	284	0.982	0.047	−0.00244	11.45
		2	292	0.990	0.016	−0.00238	12.43	283	0.981	0.024	−0.00245	11.36
		6	290	0.983	0.016	−0.00239	12.43	285	0.983	0.025	−0.00243	11.36
		12	310	0.982	0.018	−0.00223	12.06	281	0.981	0.025	−0.00247	11.53
1:4	4 °C	1	257	0.971	0.034	−0.00269	11.89	244	0.989	0.022	−0.00284	10.41
		2	263	0.980	0.028	−0.00264	10.91	237	0.986	0.025	−0.00292	10.18
		6	270	0.976	0.029	−0.00257	9.79	256	0.976	0.042	−0.00271	8.77
		12	264	0.977	0.029	−0.00263	8.88	257	0.979	0.047	−0.00270	7.31
	−21 °C	1	256	0.971	0.413	−0.00270	12.54	249	0.986	0.427	−0.00278	11.21
		2	261	0.976	0.030	−0.00266	12.14	247	0.978	0.256	−0.00281	11.06
		6	280	0.980	0.025	−0.00247	11.39	260	0.971	0.034	−0.00266	10.29
		12	270	0.970	0.033	−0.00257	11.40	256	0.974	0.032	−0.00271	10.31
	−76 °C	1	292	0.989	0.108	−0.00237	12.36	267	0.990	0.137	−0.00260	11.63
		2	300	0.987	0.018	−0.00231	12.26	272	0.995	0.019	−0.00255	11.59
		6	293	0.987	0.019	−0.00237	12.24	271	0.995	0.019	−0.00256	11.61
		12	297	0.988	0.020	−0.00234	12.22	265	0.993	0.013	−0.00262	11.71

* A_0_—the initial amount of iodine, k—decay constant [30].

**Table 3:** Parameters of carrier iodination of the iodine carriers.

Iodine Carrier	Iodine Salt Solution Concentration (mg L^−1^)
Degree of hydratation: 1:1 (carier: sulution ratio; *m*/*v*)	
Iodized KI	30.00
Iodized KIO_3_	39.00
Degree of hydratation: 1:2 (carier: sulution ratio; *m*/*v*)	
Iodized KI	15.00
Iodized KIO_3_	19.50
Degree of hydratation: 1:4 (carier: sulution ratio; *m*/*v*)	
Iodized KI	7.50
Iodized KIO_3_	9.75

## Data Availability

Not applicable.

## Supplementary Materials

The following supporting information can be downloaded at: https://www.mdpi.com/article/10.3390/molecules27103351/s1, Table S1: The color parameters of fortified pumpkin with KI; Table S2. The color parameters of fortified pumpkin with KIO_3_; Table S3: The color parameters of fortified broccoli with KI; Table S4: The color parameters of fortified broccoli with KIO_3_; Table S5: The color parameters of fortified cauliflower with KI; Table S6: The color parameters of fortified cauliflower with KIO_3_; Table S7: The color parameters of fortified carrot with KI; Table S8: The color parameters of fortified carrot with KIO_3_; Table S9: The iodine content (%) during 230 days of storage of the dried KI fortified pumpkin at various conditions (temperature, hydration, and time); Table S10: The iodine content (%) during 230 days of storage of the dried KIO_3_ fortified pumpkin at various conditions (temperature, hydration, and time); Table S11: The iodine content (%) during 230 days of storage of the dried KI fortified broccoli at various conditions (temperature, hydration, and time); Table S12: The iodine content (%) during 230 days of storage of the dried KIO_3_ fortified broccoli at various conditions (temperature, hydration, and time); Table S13: The iodine content (%) during 230 days of storage of the dried KI fortified cauliflower at various conditions (temperature, hydration, and time); Table S14: The iodine content (%) during 230 days of storage of the dried KIO_3_ fortified cauliflower at various conditions (temperature, hydration, and time); Table S15: The iodine content (%) during 230 days of storage of the dried KI fortified carrot at various conditions (temperature, hydration, and time); Table S16: The iodine content (%) during 230 days of storage of the dried KIO_3_ fortified carrot at various conditions (temperature, hydration, and time).

## Sample Availability

Samples of the compounds are available from the authors.
