# Supplementary material for: The Selection of the Optimal Impregnation Conditions of Vegetable Matrices with Iodine
Source: Molecules. 2022 May 23;27(10):3351. doi: 10.3390/molecules27103351 (PMC9144381; doi:10.3390/molecules27103351)
Supplement: Supplementary file 1 [file molecules-27-03351-s001.zip › molecules-1688513-supplementary.pdf]

Table S1. The colour parameters of fortified pumpkin with KI.

Hydration	Temperature [⁰C]	Time [hours]	The colour parameters L	a	b	C	h
hydration 1:1.	4	1	58.65	3.02	4.29	5.52	57.99
		2	58.55	3.11	5.14	5.84	56.99
		6	58.68	3.08	4.86	5.7	58.48
		12	58.74	3.05	4.79	5.68	57.51
	-21	1	58.71	3.02	4.72	5.6	57.41
		2	58.67	3.06	4.76	5.67	57.68
		6	58.24	3.07	4.74	5.65	57.04
		12	57.80	3.02	4.88	5.74	58.28
	-76	1	58.93	3.06	4.1	5.41	57.9
		2	58.68	3.21	3.79	5.41	56.41
		6	58.69	3.11	4.03	5.44	56.23
		12	58.47	3.09	4.31	5.53	57.17
hydration 1:2.	4	1	58.68	3.02	4.71	5.48	59.45
		2	58.59	3.58	3.64	5.1	55.97
		6	58.70	3.47	3.71	5.08	56.12
		12	58.52	3.02	3.59	5.07	57.01
	-21	1	58.12	3.85	4.21	5.13	59.06
		2	58.52	3.39	2.03	5.17	57.03
		6	59.65	3.49	2.21	5.12	57.71
		12	59.32	3.72	3.98	5.61	57.69
	-76	1	59.54	3.61	4.03	5.09	56.55
		2	59.51	3.73	4.21	4.99	57.03
		6	59.33	3.74	3.98	5.12	56.21
		12	59.47	3.66	3.68	5.19	56.89
hydration 1:4.	4	1	58.62	2.75	4.11	4.94	56.22
		2	58.08	3.21	3.73	4.92	55.28
		6	57.39	3.1	4.11	5.15	56.89
		12	57.61	3.17	4.02	5.12	54.63
	-21	1	57.78	3.16	3.9	5.02	55.87
		2	57.90	3.08	3.97	5.03	56.98
		6	58.41	2.85	3.89	5.21	56.82
		12	58.72	2.87	4.22	5.11	57.03
	-76	1	58.64	2.91	4.09	5.02	55.45
		2	58.68	3.02	4.03	5.03	53.18
		6	58.64	3.14	3.79	4.92	55.65
		12	58.62	2.96	4.00	5.06	55.63

Table S2. The colour parameters of fortified pumpkin with KIO_3.

Hydration	Temperature [⁰C]	Time [hours]	L	a	b	C	h
hydration 1:1.	4	1	58.45	3.01	4.3	5.21	57.99
		2	58.46	3.1	5.11	5.84	56.21
		6	58.68	3.08	4.86	5.7	56.62
		12	58.59	3.58	3.64	5.1	55.97
	-21	1	58.7	3.47	3.71	5.08	56.12
		2	58.52	3.02	3.59	5.07	57.01
		6	58.11	3.81	4.22	5.11	57.21
		12	58.26	3.29	3.98	5.01	57.09
	-76	1	59.65	3.49	3.01	5.12	57.71
		2	59.32	3.72	3.98	5.61	55.49
		6	59.54	3.21	4.03	5.09	56.55
		12	59.51	3.71	4.21	4.99	55.1
hydration 1:2.	4	1	59.33	3.74	3.98	5.12	55.23
		2	59.47	3.66	3.68	5.19	56.89
		6	58.62	2.75	4.09	4.94	56.22
		12	58.08	3.21	3.73	4.92	55.28
	-21	1	58.31	3.1	4.11	5.15	55.34
		2	57.60	3.17	4.02	5.12	54.63
		6	58.03	3.16	3.41	4.98	57.24
		12	58.07	3.01	3.97	5.03	56.98
	-76	1	58.31	3.02	4.02	5.21	56.82
		2	58.19	2.99	4.01	5.11	55.75
		6	58.71	3.01	3.99	5.02	55.45
		12	58.66	2.99	4.01	5.03	53.18
hydration 1:4.	4	1	58.54	3.03	4.03	4.92	54.69
		2	58.51	2.99	4.01	5.06	55.18
		6	58.74	3.05	4.79	5.68	57.51
		12	58.71	3.02	4.72	5.6	57.41
	-21	1	58.61	3.05	4.76	5.61	57.47
		2	58.24	3.07	4.74	5.65	57.03
		6	57.80	3.02	4.88	5.74	57.34
		12	58.93	3.06	4.1	5.41	57.67
	-76	1	58.68	3.21	3.79	5.41	57.03
		2	57.60	3.17	4.02	5.12	55.98
		6	57.70	3.15	3.90	5.02	57.69
		12	58.68	3.02	4.71	5.48	86.23

Table S3. The colour parameters of fortified broccoli with KI.

Hydration	Temperature [^{0}C]	Time [hours]	The colour parameters L	a	b	C	h
hydration 1:1.	4	1	57.31	-0.17	2.62	2.63	93.64
		2	57.17	-0.20	2.69	2.70	94.29
		6	57.15	-0.23	2.66	2.67	94.91
		12	57.12	-0.19	2.61	2.62	94.23
	-21	1	57.03	0.10	2.66	2.67	94.88
		2	57.16	-0.20	2.65	2.66	94.39
		6	57.83	0.01	2.22	2.22	89.63
		12	57.82	0.07	2.13	2.13	92.41
	-76	1	57.18	0.21	2.61	1.99	92.38
		2	57.77	0.30	1.87	2.21	90.89
		6	57.64	0.09	1.98	2.03	88.56
		12	57.65	0.25	2.27	2.12	90.12
hydration 1:2.	4	1	56.18	0.22	2.66	2.67	90.23
		2	55.77	-0.15	2.98	2.61	92.70
		6	57.12	-0.17	3.11	2.12	93.08
		12	56.78	-0.08	3.08	2.09	91.57
	-21	1	56.16	-0.05	2.97	2.97	90.88
		2	56.77	-0.05	2.99	2.37	91.98
		6	56.98	-0.13	3.31	2.56	92.32
		12	56.45	-0.25	2.98	2.26	90.21
	-76	1	57.45	-0.26	3.04	2.48	93.14
		2	57.12	-0.16	3.02	3.32	92.68
		6	57.23	-0.13	3.34	2.21	92.28
		12	56.45	-0.19	2.98	2.20	91.89
hydration 1:4.	4	1	57.97	0.30	2.76	2.24	91.45
		2	57.96	0.14	3.04	2.39	90.89
		6	57.90	0.13	3.02	2.40	90.78
		12	56.28	0.17	2.99	2.35	91.45
	-21	1	57.75	0.42	3.05	2.06	91.34
		2	57.88	0.23	3.01	2.29	92.45
		6	56.45	-0.17	3.02	2.03	92.45
		12	56.89	-0.08	2.98	2.37	92.45
	-76	1	57.23	0.12	2.65	2.46	91.78
		2	57.12	-0.08	2.78	2.69	92.07
		6	56.89	-0.05	3.12	2.25	93.71
		12	56.45	0.12	3.16	2.21	92.45

Table S4. The colour parameters of fortified broccoli with KIO_3.

Hydration	Temperature [⁰C]	Time [hours]	The colour parameters L	a	b	C	h
hydration 1:1.	4	1	57.29	-0.17	2.61	2.61	94.01
		2	57.09	-0.20	2.69	2.72	90.23
		6	57.02	-0.23	2.66	3.01	92.45
		12	57.12	-0.19	2.61	2.59	91.78
	-21	1	56.65	0.10	2.66	2.71	92.07
		2	57.03	-0.12	2.65	2.70	93.71
		6	57.83	-0.15	2.22	2.31	92.45
		12	57.82	-0.11	2.13	2.09	92.70
	-76	1	57.18	-0.13	2.61	2.04	93.08
		2	57.77	-0.13	1.87	2.22	91.57
		6	57.64	-0.21	1.98	2.13	90.88
		12	57.65	-0.12	2.27	2.09	90.12
hydration 1:2.	4	1	56.18	0.11	2.66	2.19	91.29
		2	56.89	-0.08	2.98	2.12	90.23
		6	57.23	0.12	2.65	2.21	91.03
		12	57.12	-0.09	2.51	2.22	90.32
	-21	1	56.89	-0.23	2.49	2.09	92.31
		2	56.45	0.08	2.44	2.03	91.03
		6	55.77	-0.15	2.98	2.12	90.45
		12	57.12	-0.17	2.23	2.21	91.23
	-76	1	56.78	-0.08	2.51	2.19	90.31
		2	56.16	-0.05	2.97	2.54	90.34
		6	56.65	-0.05	2.37	2.49	91.98
		12	56.78	-0.13	2.31	2.16	92.32
hydration 1:4.	4	1	57.03	-0.25	2.98	2.34	91.03
		2	57.29	-0.26	2.12	3.45	92.03
		6	57.03	-0.16	2.34	3.01	91.56
		12	56.98	-0.13	2.56	2.21	90.45
	-21	1	57.24	-0.19	2.33	3.12	91.89
		2	57.12	-0.19	2.76	2.56	90.65
		6	57.36	-0.11	2.45	1.12	90.89
		12	56.98	0.13	2.31	3.03	91.78
	-76	1	57.03	0.17	2.99	2.21	91.45
		2	57.75	0.09	2.47	2.06	91.34
		6	57.88	0.12	2.49	3.02	92.45
		12	56.45	-0.16	2.64	2.05	91.38

Table S5. The colour parameters of fortified cauliflower with KI..

Hydration	Temperature [^{0}C]	Time [hours]	L	a	b	C	h
hydration 1:1.	4	1	57.54	0.28	3.67	3.68	87.46
		2	56.84	0.22	3.87	3.88	87.67
		6	54.57	0.29	4.00	4.01	87.69
		12	56.27	0.14	3.85	3.86	87.95
	-21	1	56.33	0.22	3.80	3.81	89.54
		2	56.31	0.23	3.84	3.85	90.13
		6	60.52	0.42	3.60	3.62	83.30
		12	60.25	0.37	3.74	3.76	84.38
	-76	1	60.14	0.39	3.70	3.72	83.96
		2	59.94	0.31	3.72	3.73	85.21
		6	59.98	0.38	3.69	3.71	90.26
		12	60.17	0.37	3.69	3.71	84.20
hydration 1:2.	4	1	60.37	0.62	3.43	3.49	88.56
		2	60.61	0.59	3.60	3.65	80.72
		6	60.52	0.55	3.61	3.65	81.37
		12	60.63	0.45	3.64	3.67	82.91
	-21	1	60.67	0.52	3.58	3.61	81.74
		2	60.56	0.55	3.57	3.61	81.30
		6	58.06	-0.15	4.53	4.53	91.09
		12	55.09	-0.16	4.30	3.98	90.23
	-76	1	54.98	-0.20	4.27	4.27	89.45
		2	54.81	-0.19	4.24	4.24	89.45
		6	54.97	-0.28	4.26	4.06	89.45
		12	55.58	-0.20	4.32	4.32	90.23
hydration 1:4.	4	1	59.93	-0.08	3.87	3.87	91.21
		2	60.93	0.18	3.47	3.48	87.00
		6	60.57	0.03	3.64	3.64	89.47
		12	60.28	-0.02	3.78	3.78	90.31
	-21	1	60.16	-0.05	3.82	3.82	90.71
		2	60.37	0.01	3.72	3.72	89.74
		6	60.58	0.59	3.57	3.62	88.45
		12	60.91	0.32	4.01	4.03	88.78
	-76	1	60.38	0.18	4.21	3.99	87.49
		2	59.37	0.05	4.41	4.41	89.33
		6	58.99	0.07	4.46	4.06	89.06
		12	60.05	0.24	4.13	4.15	86.38

Table S6. The colour parameters of fortified cauliflower with KIO_3.

Hydration	Temperature [⁰C]	Time [hours]	L	a	b	C	h
hydration 1:1.	4	1	57.45	0.26	3.66	3.66	90.21
		2	57.65	0.25	4.21	3.87	89.89
		6	55.38	-0.11	3.98	3.98	87.00
		12	56.24	-0.13	3.75	3.90	87.45
	-21	1	56.33	-0.12	3.45	3.79	90.31
		2	56.31	0.12	3.61	3.86	90.71
		6	55.45	-0.13	3.54	3.63	89.74
		12	56.23	-0.17	3.45	3.70	88.45
	-76	1	56.48	-0.18	3.51	3.70	88.78
		2	56.87	0.18	3.61	3.69	87.49
		6	57.23	0.03	3.58	3.55	89.33
		12	57.23	-0.02	3.73	3.69	89.06
hydration 1:2.	4	1	57.48	-0.04	3.21	3.52	86.38
		2	57.69	0.01	3.61	3.54	85.64
		6	56.98	0.59	3.45	3.66	85.49
		12	56.48	0.32	4.24	3.49	82.91
	-21	1	55.98	0.52	4.03	3.29	89.45
		2	57.67	0.55	4.32	3.79	88.45
		6	57.46	-0.15	3.87	3.99	90.23
		12	56.03	-0.16	3.47	4.01	90.23
	-76	1	56.89	-0.12	3.64	4.02	89.45
		2	57.03	-0.19	3.78	3.98	89.45
		6	56.48	-0.28	3.82	3.79	89.45
		12	56.36	-0.20	3.72	4.32	90.23
hydration 1:4.	4	1	55.98	0.21	3.42	3.64	89.61
		2	56.23	0.18	3.51	4.01	87.00
		6	56.48	0.12	3.49	3.79	89.47
		12	56.98	-0.02	3.77	4.02	90.31
	-21	1	56.77	-0.05	3.73	3.77	90.71
		2	56.98	0.01	3.49	3.69	89.74
		6	56.12	0.59	3.97	3.78	88.45
		12	57.03	0.32	3.99	3.78	88.78
	-76	1	57.09	0.18	3.49	4.01	87.49
		2	55.36	0.05	3.21	3.65	89.33
		6	58.99	0.07	3.98	3.70	89.06
		12	56.78	0.24	4.01	3.70	89.45

Table S7. The colour parameters of fortified carrot with KI..

Hydration	Temperature [⁰C]	Time [hours]	L	a	b	C	h
hydration 1:1.	4	1	58.34	2.64	3.01	4.99	50.01
		2	58.28	3.02	3.05	5.01	50.45
		6	58.33	2.76	2.92	5.02	49.52
		12	58.28	3.01	2.86	4.99	49.71
	-21	1	58.08	2.60	3.17	4.99	50.56
		2	58.26	2.67	2.98	5.21	50.03
		6	58.73	2.98	3.06	5.21	50.26
		12	58.64	3.01	3.08	4.98	50.21
	-76	1	58.43	3.39	3.10	4.99	50.39
		2	58.11	3.47	3.12	5.21	50.45
		6	58.22	3.51	3.10	5.75	50.31
		12	58.43	3.48	3.15	5.64	50.12
hydration 1:2.	4	1	58.30	3.56	2.93	5.22	50.02
		2	58.42	3.42	3.09	5.31	50.01
		6	58.29	3.66	2.82	4.99	50.11
		12	58.17	3.68	3.01	4.61	49.31
	-21	1	58.16	3.71	2.78	4.63	49.74
		2	58.27	3.61	2.88	4.62	50.02
		6	58.64	2.85	2.98	4.65	50.01
		12	58.93	3.04	3.01	4.54	49.98
	-76	1	59.01	3.12	3.31	4.98	49.87
		2	58.90	3.27	3.13	4.53	49.98
		6	58.80	3.34	2.97	4.47	50.11
		12	58.86	3.12	3.09	4.55	50.09
hydration 1:4.	4	1	58.42	3.42	3.09	5.31	50.01
		2	58.29	3.66	2.82	4.99	50.11
		6	58.17	3.68	3.01	4.61	49.31
		12	58.33	2.76	2.92	5.02	49.52
	-21	1	58.28	3.01	2.86	4.99	49.71
		2	58.01	2.65	3.17	5.01	50.54
		6	58.31	2.78	2.98	4.98	50.03
		12	59.01	3.08	3.31	4.96	50.78
	-76	1	58.90	3.27	3.13	4.53	49.98
		2	58.33	2.89	2.92	4.87	50.19
		6	58.28	3.01	2.86	4.99	49.71
		12	58.08	3.02	3.17	5.02	50.56

Table S8. The colour parameters of fortified carrot with KIO_3.

Hydration	Temperature [⁰C]	Time [hours]	L	a	b	C	h
hydration 1:1.	4	1	58.21	2.66	3.02	4.99	49.98
		2	59.32	2.69	3.01	5.01	50.45
		6	58.25	3.01	3.00	5.02	49.52
		12	58.36	3.02	3.00	4.99	49.71
	-21	1	59.64	2.78	2.98	4.99	5051.00
		2	58.26	2.89	3.03	5.21	50.23
		6	58.73	2.98	2.98	5.21	50.03
		12	58.64	2.87	2.99	4.98	49.89
	-76	1	58.33	3.02	3.12	4.99	49.78
		2	58.11	2.98	2.87	5.21	50.41
		6	58.27	3.21	2.76	5.75	50.31
		12	58.64	3.12	3.09	5.64	50.12
hydration 1:2.	4	1	58.76	3.98	3.11	5.22	50.02
		2	59.01	3.42	3.10	5.31	50.01
		6	58.90	3.66	3.09	4.99	50.03
		12	58.80	3.68	2.78	4.61	49.35
	-21	1	58.86	3.71	3.11	4.63	49.78
		2	58.41	3.61	3.02	4.62	50.02
		6	58.30	2.76	3.02	4.65	50.01
		12	58.17	3.01	2.69	4.54	49.98
	-76	1	58.33	2.65	3.29	4.98	49.87
		2	58.28	2.78	2.97	4.53	49.97
		6	58.01	3.08	2.92	4.47	49.23
		12	58.31	3.22	3.09	4.55	49.69
hydration 1:4.	4	1	58.78	2.89	2.91	5.31	5.03
		2	58.12	3.01	2.82	4.99	50.11
		6	58.33	3.04	2.99	4.61	49.31
		12	58.28	3.12	3.01	5.02	49.78
	-21	1	58.08	3.27	2.99	4.99	49.71
		2	59.09	3.34	3.12	5.01	50.54
		6	59.02	3.12	3.02	4.98	50.21
		12	58.30	3.42	3.31	4.96	49.69
	-76	1	58.42	3.66	3.00	4.53	49.98
		2	58.29	3.68	2.99	4.87	50.09
		6	58.17	2.95	2.86	4.99	49.71
		12	58.16	3.02	3.06	5.02	50.21

Table S9. The iodine content (%) during 230 days storage of the dried KI fortified pumpkin at various conditions (temperature, hydration and time).

Hydration	Temperature [^{0}C]	Time [hours]	Iodine content [%] Storage [days] 30	60	90	120	150	180	230
hydration 1:1.	4	1	96.67	91.77	89.68	86.29	83.88	80.38	72.74
		2	96.68	92.10	89.82	85.44	83.77	80.92	71.41
		6	95.61	89.70	88.91	84.88	82.50	80.01	72.09
		12	94.36	90.15	87.37	86.01	81.73	77.57	70.11
	-21	1	97.56	92.57	91.21	88.35	84.77	82.26	73.94
		2	97.54	91.68	91.35	87.92	86.03	82.30	74.45
		6	96.45	90.57	89.13	86.38	85.54	81.66	73.76
		12	96.04	90.15	88.57	85.57	83.76	81.70	73.85
	-76	1	97.05	93.10	91.15	88.64	84.97	81.68	78.33
		2	97.03	93.10	91.10	88.60	84.75	81.65	78.56
		6	97.12	92.98	91.09	88.23	84.69	81.89	78.03
		12	97.01	93.21	91.23	88.67	84.99	81.28	78.29
hydration 1:2.	4	1	97.56	92.57	91.21	88.35	84.77	82.26	73.90
		2	97.54	91.68	91.35	87.92	86.03	82.31	74.45
		6	96.45	90.57	89.13	86.38	85.51	81.66	73.76
		12	96.04	90.16	88.57	85.57	83.76	81.70	73.85
	-21	1	97.56	92.57	91.21	88.35	84.77	82.26	73.94
		2	97.99	91.68	91.35	87.92	86.03	82.30	74.45
		6	97.23	90.57	89.13	86.38	85.54	81.66	73.76
		12	96.98	90.15	88.57	85.57	83.76	81.70	73.85
	-76	1	97.11	93.45	91.16	88.69	84.78	81.29	78.25
		2	97.56	93.45	91.24	88.73	84.59	81.56	78.69
		6	97.23	93.09	91.03	88.56	84.23	81.79	78.46
		12	97.23	93.65	91.19	88.79	84.97	81.82	78.69
hydration 1:4.	4	1	96.67	91.77	89.68	86.29	83.88	80.38	72.74
		2	96.68	92.10	89.82	85.44	83.77	80.92	71.41
		6	95.61	89.70	88.91	84.88	82.50	80.01	72.09
		12	94.36	90.15	87.37	86.01	81.73	77.57	70.11
	-21	1	97.56	92.57	91.21	88.35	84.77	82.26	73.94
		2	97.54	91.68	91.35	87.92	86.03	82.30	74.45
		6	96.45	90.57	89.13	86.38	85.54	81.66	73.76
		12	96.04	90.15	88.57	85.57	83.76	81.70	73.85
	-76	1	97.05	93.10	91.15	88.64	84.97	81.68	78.33
		2	97.03	93.10	91.10	88.60	84.75	81.65	78.56
		6	97.12	92.98	91.09	88.23	84.69	81.89	78.03
		12	97.01	93.21	91.23	88.67	84.99	81.28	78.29

Table S10. The iodine content (%) during 230 days storage of the dried KIO_3 fortified pumpkin at various conditions (temperature, hydration and time).

Hydration	Temperature [ºC]	Time [hours]	Iodine content [%]						
			Storage [days]						
			30	60	90	120	150	180	230
hydration 1:1.	4	1	97.87	95.05	93.42	92.19	88.34	85.03	81.61
		2	97.88	95.13	93.25	92.39	88.96	85.12	81.03
		6	96.80	94.91	92.90	91.29	88.03	84.56	81.11
		12	96.37	94.94	91.69	91.03	87.23	84.03	80.89
	-21	1	97.23	95.23	94.41	92.36	89.26	84.98	80.03
		2	97.56	95.29	94.24	92.56	88.20	85.12	81.38
		6	97.69	95.03	94.69	92.77	88.37	85.09	81.26
		12	97.26	95.45	94.78	92.03	88.21	85.77	81.80
	-76	1	98.26	95.98	94.01	92.32	88.65	84.50	81.20
		2	98.31	95.45	94.01	92.41	88.03	84.61	81.31
		6	98.19	95.99	94.12	92.09	88.15	84.60	81.29
		12	98.29	95.79	94.12	92.19	88.19	84.41	81.19
hydration 1:2.	4	1	97.54	95.21	93.12	90.53	88.12	83.82	76.21
		2	97.77	95.55	93.25	89.68	88.01	84.36	74.85
		6	96.80	93.22	92.42	89.19	86.88	83.52	75.60
		12	95.61	93.03	90.25	89.69	85.41	80.45	72.98
	-21	1	98.55	95.45	94.12	92.03	88.45	85.14	80.23
		2	98.77	94.56	94.23	91.60	89.71	85.18	80.45
		6	97.65	93.45	92.01	90.06	89.22	84.54	80.31
		12	97.99	93.06	91.46	90.65	87.44	84.58	79.81
	-76	1	98.26	95.98	94.03	92.32	88.56	84.55	81.19
		2	98.44	95.75	94.03	92.45	88.23	84.32	81.22
		6	98.61	95.29	94.11	92.45	88.95	84.56	81.23
		12	98.38	95.73	94.03	92.03	88.79	84.35	81.26
hydration 1:4.	4	1	97.87	94.65	92.56	89.97	87.56	83.26	75.65
		2	97.88	94.99	92.69	89.12	87.45	83.80	74.29
		6	96.80	92.59	91.79	88.56	86.25	82.89	74.97
		12	95.56	93.03	90.25	89.69	85.41	80.45	72.98
	-21	1	98.76	95.45	94.12	91.98	88.03	84.23	76.82
		2	98.77	94.56	94.23	91.56	88.23	84.39	77.36
		6	97.65	93.45	92.01	90.56	88.32	84.54	76.64
		12	97.26	93.05	91.45	89.25	87.45	84.58	76.73
	-76	1	98.25	95.98	94.03	92.32	88.65	84.56	81.21
		2	98.45	95.45	94.03	92.45	88.01	84.28	81.20
		6	98.41	95.99	94.11	92.45	88.39	84.79	81.64
		12	98.03	95.79	94.09	92.03	88.35	84.36	81.03

Table S11. The iodine content (%) during 230 days storage of the dried KI fortified broccoli at various conditions (temperature, hydration and time).

Hydration	Temperature [°C]	Time [hours]	Iodine content [%] Storage [days] 30	60	90	120	150	180	230
hydration 1:1.	4	1	97.54	93.00	93.03	87.95	84.03	81.26	78.89
		2	97.00	95.98	90.68	87.96	83.98	81.37	78.65
		6	96.03	92.03	91.99	87.42	83.78	80.56	77.56
		12	96.19	91.89	89.78	86.95	82.76	80.03	77.43
	-21	1	97.51	93.00	90.79	87.98	82.89	81.98	78.56
		2	97.03	92.56	90.99	88.12	83.81	81.56	78.69
		6	97.12	92.78	90.93	87.95	83.79	82.56	78.45
		12	96.87	92.83	89.00	87.93	83.59	82.12	78.69
	-76	1	96.54	93.00	90.56	88.03	83.00	82.37	78.19
		2	96.48	92.79	90.79	87.69	82.98	82.19	78.98
		6	96.37	92.98	90.65	88.02	82.97	82.64	78.46
		12	96.87	92.94	90.89	87.73	83.11	81.98	78.69
hydration 1:2.	4	1	97.55	92.50	91.03	88.30	85.48	82.26	73.89
		2	97.51	91.60	90.98	88.03	85.21	82.31	74.50
		6	96.40	90.02	88.45	85.89	84.97	81.66	73.50
		12	96.01	89.59	87.79	85.01	82.89	81.70	72.89
	-21	1	97.12	92.03	90.78	87.79	84.89	82.26	73.02
		2	97.45	91.25	90.76	87.12	84.21	82.30	73.06
		6	97.03	90.03	89.00	86.03	83.56	81.66	73.54
		12	96.24	90.01	88.03	85.09	82.98	81.70	73.01
	-76	1	97.10	92.89	90.59	88.02	83.78	81.29	77.89
		2	97.12	92.99	90.12	87.73	83.45	81.56	77.56
		6	97.39	92.71	90.79	87.69	83.45	81.79	77.39
		12	97.02	92.75	90.43	88.02	83.89	81.82	77.67
hydration 1:4.	4	1	96.66	91.76	89.60	86.30	83.87	80.12	72.45
		2	96.69	92.11	89.80	85.41	83.50	80.11	71.03
		6	96.03	89.50	88.92	84.78	82.41	79.25	71.23
		12	94.35	90.15	87.35	86.01	81.25	77.00	69.78
	-21	1	97.55	92.50	91.20	88.24	84.72	81.25	74.03
		2	97.50	91.50	91.34	87.85	86.00	81.45	73.49
		6	96.45	90.56	89.10	86.21	85.21	81.02	73.26
		12	96.03	90.14	88.56	85.61	83.61	80.59	73.03
	-76	1	97.06	93.08	91.10	88.50	84.02	81.45	78.03
		2	97.03	93.11	91.11	88.48	84.89	81.38	77.99
		6	97.09	93.12	91.08	88.39	84.91	81.25	77.89
		12	97.12	93.21	91.05	88.45	84.79	81.19	78.01

Table S12. The iodine content (%) during 230 days storage of the dried KIO_3 fortified broccoli at various conditions (temperature, hydration and time).

Hydration	Temperature [ºC]	Time [hours]	Iodine content [%] Storage [days] 30	60	90	120	150	180	230
hydration 1:1.	4	1	97.80	95.01	93.01	91.90	88.50	83.25	80.78
		2	97.77	95.00	94.98	91.98	88.42	83.36	80.89
		6	96.42	94.25	92.45	90.89	87.89	83.09	80.03
		12	96.03	93.98	91.60	90.78	86.97	82.97	79.03
	-21	1	97.02	95.01	93.78	92.03	88.97	83.29	81.04
		2	97.03	94.93	93.89	92.01	88.99	82.19	80.79
		6	97.11	94.94	93.78	92.34	88.76	83.25	81.11
		12	96.97	94.83	93.45	92.09	88.62	83.06	80.78
	-76	1	97.01	94.98	91.12	91.99	88.96	83.30	80.91
		2	96.98	95.01	93.11	91.89	89.03	83.31	81.09
		6	97.11	95.03	93.98	91.99	88.81	83.11	80.67
		12	96.89	94.93	93.78	91.78	88.97	83.35	80.56
hydration 1:2.	4	1	97.50	95.20	92.80	90.00	88.02	83.50	76.03
		2	97.23	95.03	92.45	89.00	87.56	83.90	74.00
		6	96.56	93.02	91.45	88.78	86.72	83.00	74.95
		12	95.45	92.89	89.78	88.21	84.78	80.01	72.00
	-21	1	98.45	95.00	93.89	91.88	88.03	84.52	79.89
		2	98.45	94.03	93.82	91.56	88.94	84.78	79.91
		6	97.00	94.03	91.45	91.03	88.97	84.09	79.38
		12	97.03	92.78	91.00	91.09	87.68	84.01	79.50
	-76	1	97.39	95.00	93.75	90.78	87.39	84.00	80.23
		2	97.89	95.14	93.67	91.56	87.25	83.75	80.98
		6	97.91	94.78	93.64	91.45	87.39	83.21	80.29
		12	97.68	94.38	93.50	91.29	87.25	83.56	80.78
hydration 1:4.	4	1	97.86	97.64	92.50	89.70	87.50	83.20	75.03
		2	97.88	94.89	92.49	88.78	87.03	82.41	74.01
		6	96.89	92.50	91.40	87.89	85.74	81.79	73.89
		12	95.41	93.00	89.89	87.79	84.79	79.29	72.01
	-21	1	98.70	95.40	94.10	90.56	87.79	83.08	76.23
		2	98.70	94.50	93.89	90.41	87.01	83.12	77.30
		6	97.60	93.30	91.99	91.03	87.00	82.99	77.29
		12	97.25	93.21	91.60	89.12	86.77	82.48	73.45
	-76	1	98.19	95.02	93.78	91.89	88.01	83.29	80.78
		2	98.22	95.00	93.50	91.78	87.79	83.11	80.45
		6	98.21	94.89	93.59	91.99	87.91	83.15	80.23
		12	98.19	94.99	93.51	91.68	87.79	83.00	80.03

Table S13. The iodine content (%) during 230 days storage of the dried KI fortified cauliflower at various conditions (temperature, hydration and time).

Hydration	Temperature [⁰C]	Time [hours]	Iodine content [%]						
			Storage [days]						
			30	60	90	120	150	180	230
hydration 1:1.	4	1	97.50	93.02	93.00	87.80	83.98	81.20	78.80
		2	97.03	93.12	92.52	87.90	83.89	81.19	78.60
		6	96.12	92.31	92.89	87.40	83.50	80.59	77.90
		12	96.15	91.56	89.95	87.00	82.98	80.06	77.06
	-21	1	97.20	92.98	90.50	87.56	82.90	81.90	78.60
		2	97.05	92.49	90.45	87.98	82.78	81.86	78.60
		6	97.09	92.82	90.80	87.73	82.69	81.94	78.21
		12	96.95	92.90	90.12	87.88	82.54	82.02	78.20
	-76	1	96.45	92.89	90.20	88.12	82.89	82.03	78.25
		2	96.23	92.89	89.99	88.03	82.65	82.01	78.61
		6	96.35	93.05	89.92	87.92	83.02	82.09	78.45
		12	96.45	92.90	90.45	87.96	82.61	81.96	78.03
hydration 1:2.	4	1	97.54	90.51	91.03	88.21	85.50	82.30	73.90
		2	97.50	90.48	90.75	88.01	85.20	82.30	74.45
		6	96.50	89.98	88.12	85.89	84.90	81.70	72.49
		12	96.08	89.60	87.60	85.03	82.90	81.25	72.90
	-21	1	97.10	92.06	90.50	87.60	84.70	82.30	75.69
		2	97.30	92.09	90.60	87.10	84.19	82.25	75.69
		6	97.09	90.12	89.25	86.69	83.60	82.03	76.03
		12	96.25	91.26	88.11	86.25	83.69	81.98	75.03
	-76	1	97.10	92.89	91.03	86.56	83.50	81.26	76.35
		2	97.09	92.56	90.10	87.98	83.69	81.11	76.69
		6	97.20	92.45	90.60	87.69	83.45	81.03	76.78
		12	97.09	92.19	90.21	87.99	83.90	82.03	76.98
hydration 1:4.	4	1	96.66	91.78	89.61	86.29	83.90	79.98	72.40
		2	96.70	92.10	89.60	85.64	83.51	79.99	71.03
		6	96.04	89.54	88.93	84.90	82.40	79.25	71.00
		12	94.31	90.14	87.41	86.01	82.30	74.03	69.69
	-21	1	97.50	92.51	91.21	88.26	84.56	80.90	74.01
		2	97.51	91.48	91.32	87.46	85.03	80.56	73.30
		6	96.46	90.58	89.10	86.30	85.03	80.78	73.31
		12	96.01	90.21	88.98	85.60	84.25	80.12	72.98
	-76	1	97.05	93.01	91.09	88.51	83.21	81.03	77.89
		2	97.03	93.10	91.22	88.47	84.75	81.11	77.98
		6	97.09	93.10	91.26	88.43	84.92	81.02	77.75
		12	97.05	93.09	91.09	88.51	83.54	81.03	77.35

Table S14. The iodine content (%) during 230 days storage of the dried KIO_3 fortified cauliflower at various conditions (temperature, hydration and time).

Hydration	Temperature [⁰C]	Time [hours]	Iodine content [%] Storage [days] 30	60	90	120	150	180	230
hydration 1:1.	4	1	97.81	95.02	93.12	91.91	88.52	84.80	80.78
		2	97.75	94.89	93.98	91.99	88.30	84.65	81.05
		6	97.69	94.56	93.12	90.90	87.90	84.05	80.95
		12	96.52	94.69	91.45	90.56	87.03	83.70	80.03
	-21	1	97.00	95.00	93.70	92.04	88.90	84.60	81.02
		2	97.25	94.80	93.64	92.03	88.85	84.09	81.25
		6	97.13	94.79	93.70	90.30	88.70	84.01	81.60
		12	97.13	94.80	93.56	90.10	88.59	83.98	81.59
	-76	1	97.03	94.86	92.98	90.80	88.90	84.12	81.53
		2	97.69	95.23	93.25	91.90	88.95	83.78	81.46
		6	97.45	95.03	93.65	91.50	89.03	84.23	81.70
		12	97.13	95.19	93.60	91.52	89.05	84.06	81.29
hydration 1:2.	4	1	97.49	95.21	92.81	90.03	88.05	83.45	76.12
		2	97.26	95.98	92.60	89.11	87.60	83.80	74.02
		6	96.60	93.21	91.51	88.62	86.70	83.01	73.90
		12	95.50	92.90	89.80	88.15	84.80	79.98	72.65
	-21	1	98.40	95.09	93.90	91.87	88.06	84.51	79.90
		2	98.39	97.12	93.71	91.55	88.72	84.50	79.89
		6	97.12	94.16	91.50	91.45	88.88	84.02	79.52
		12	97.09	92.90	92.03	91.23	87.70	81.03	79.40
	-76	1	97.40	95.32	93.60	91.12	87.40	83.89	80.21
		2	97.23	95.29	93.54	91.09	87.21	83.98	79.56
		6	97.19	95.60	93.67	91.03	87.30	84.03	79.89
		12	97.21	95.12	93.45	91.12	87.35	83.70	79.98
hydration 1:4.	4	1	97.88	95.69	92.48	89.60	87.60	83.21	75.00
		2	97.87	94.89	92.40	88.90	87.06	82.40	74.03
		6	96.80	92.45	91.35	87.56	85.78	81.80	73.80
		12	95.50	92.09	89.90	87.45	84.80	79.03	72.03
	-21	1	98.71	95.02	94.11	90.45	87.80	83.01	76.03
		2	98.69	94.25	93.99	90.23	87.02	82.98	76.25
		6	97.61	93.25	92.03	90.09	87.02	83.01	76.98
		12	97.23	93.06	93.95	89.11	86.59	82.64	75.98
	-76	1	98.20	94.98	93.45	90.98	87.87	83.12	80.90
		2	98.15	94.78	93.24	90.89	87.95	83.16	80.56
		6	98.34	94.01	93.09	91.56	87.35	83.02	80.45
		12	98.06	94.03	93.18	90.36	87.02	83.09	80.78

Table S15. The iodine content (%) during 230 days storage of the dried KI fortified carrot at various conditions (temperature, hydration and time).

Hydration	Temperature [°C]	Time [hours]	Iodine content [%]						
			Storage [days]						
			30	60	90	120	150	180	230
hydration 1:1.	4	1	96.61	92.13	92.11	86.91	83.09	80.31	77.40
		2	96.15	92.23	91.63	87.01	83.00	80.30	77.20
		6	95.20	91.42	91.89	86.51	82.61	79.70	76.50
		12	95.21	90.67	89.68	86.11	82.09	79.17	75.63
	-21	1	96.29	92.09	89.61	86.67	82.01	81.01	77.20
		2	96.20	91.61	89.60	87.09	81.80	80.97	77.17
		6	96.20	91.93	89.91	86.85	81.80	81.13	76.80
		12	96.06	91.90	89.23	86.99	81.66	81.13	76.77
	-76	1	95.50	92.00	89.31	87.23	82.00	81.14	77.05
		2	95.31	92.10	89.10	87.14	81.76	81.12	76.89
		6	95.49	92.09	89.03	87.03	82.13	81.20	77.02
		12	95.60	92.01	89.56	87.07	81.72	81.07	77.03
hydration 1:2.	4	1	96.64	89.61	90.13	87.31	84.60	81.40	73.01
		2	96.61	89.58	89.85	87.11	84.30	81.40	73.02
		6	95.62	89.08	87.21	84.99	84.01	80.80	72.03
		12	95.20	88.70	86.65	84.13	82.00	80.35	71.45
	-21	1	96.27	91.16	89.61	86.70	83.80	81.40	74.79
		2	96.40	91.19	89.70	86.21	83.30	81.35	74.79
		6	96.19	89.22	88.35	85.80	82.69	81.13	75.13
		12	95.35	90.36	87.21	85.35	82.79	81.08	74.13
	-76	1	96.21	91.99	90.13	85.70	82.60	80.40	75.41
		2	96.08	91.66	89.20	87.10	82.79	80.12	75.20
		6	96.12	91.55	89.70	87.02	82.55	80.29	75.13
		12	96.25	91.30	89.31	87.01	83.00	80.38	75.69
hydration 1:4.	4	1	95.68	90.80	88.63	85.30	82.56	79.00	70.40
		2	95.72	91.12	88.62	84.66	81.98	79.01	69.07
		6	95.06	88.56	87.95	83.92	81.45	78.27	69.04
		12	93.30	89.12	86.40	85.03	80.10	73.02	67.73
	-21	1	96.52	91.53	90.23	87.28	83.98	79.92	72.05
		2	96.53	90.50	90.34	86.48	84.05	79.58	71.34
		6	95.48	89.60	88.12	85.31	84.04	79.80	71.35
		12	95.03	89.23	88.00	84.90	83.27	79.14	71.02
	-76	1	96.07	92.03	90.11	87.53	82.23	80.05	75.94
		2	96.05	92.12	90.20	87.49	83.77	80.13	75.98
		6	96.11	92.12	90.28	87.45	83.90	80.04	75.87
		12	96.07	92.11	90.11	87.53	82.56	80.05	75.62

Table S16. The iodine content (%) during 230 days storage of the dried KIO_3 fortified carrot at various conditions (temperature, hydration and time).

Hydration	Temperature [°C]	Time [hours]	Iodine content [%] Storage [days] 30	60	90	120	150	180	230
hydration 1:1.	4	1	97.10	94.30	92.41	91.21	87.82	84.10	78.45
		2	97.09	94.20	92.28	91.29	87.60	83.95	78.29
		6	97.26	93.80	92.12	90.20	87.20	83.35	78.09
		12	96.01	93.95	91.03	89.86	86.33	83.02	77.23
	-21	1	97.03	94.28	92.59	91.34	88.21	83.90	78.99
		2	96.55	94.12	92.94	91.33	88.15	83.78	78.45
		6	96.43	94.10	93.05	89.60	88.00	83.31	78.38
		12	96.43	94.11	92.86	89.40	87.80	83.28	78.15
	-76	1	96.98	94.32	92.28	90.10	86.45	82.20	78.45
		2	96.99	94.29	92.55	90.38	86.19	82.38	78.32
		6	96.75	94.31	92.49	90.80	86.56	82.01	78.02
		12	96.43	94.11	92.90	91.03	86.49	82.79	79.23
hydration 1:2.	4	1	96.69	94.41	92.01	89.23	87.25	82.65	74.67
		2	96.46	95.18	91.80	88.31	86.80	83.00	72.56
		6	95.80	92.41	90.71	87.81	85.90	82.21	72.45
		12	94.71	92.10	89.00	87.36	84.00	80.20	71.21
	-21	1	97.60	94.29	92.09	91.07	87.26	83.71	78.45
		2	97.59	96.32	92.91	90.75	87.92	83.70	78.44
		6	96.32	93.25	90.70	90.65	88.08	83.22	78.07
		12	96.29	92.10	91.23	90.44	86.90	80.23	77.95
	-76	1	96.61	94.50	93.11	90.32	86.61	83.09	78.70
		2	96.50	94.50	92.80	90.32	86.30	83.18	78.60
		6	96.49	94.80	92.60	90.36	86.49	83.23	78.32
		12	96.62	94.60	92.55	90.29	86.45	82.90	78.39
hydration 1:4.	4	1	96.92	94.73	91.52	88.64	86.64	82.25	73.10
		2	96.91	93.93	91.40	87.94	86.10	81.44	73.02
		6	95.84	91.49	90.39	86.60	84.82	80.84	71.78
		12	94.50	91.13	88.94	86.49	83.84	78.07	70.12
	-21	1	97.75	94.06	93.15	89.49	86.84	82.05	74.11
		2	97.73	93.30	93.03	89.27	86.06	82.02	74.33
		6	96.66	92.29	91.07	89.13	86.06	82.05	75.06
		12	96.27	92.10	92.99	88.15	85.63	81.68	74.06
	-76	1	97.20	94.02	92.50	90.10	86.91	82.16	78.21
		2	97.19	93.30	92.28	89.95	86.99	82.20	78.72
		6	97.38	93.02	92.13	90.03	86.41	82.11	78.12
		12	97.10	93.10	92.22	90.03	86.20	82.20	78.45
